# Interfacial Organization and Structural Changes in Model Lung Surfactants Induced by Methylxanthines

**DOI:** 10.1021/acs.langmuir.6c01921

**Published:** 2026-06-04

**Authors:** Wiktoria Kołomyjska, Michalina Zaborowska-Mazurkiewicz, Philippe Fontaine, Dorota Matyszewska

**Affiliations:** † 49605 University of Warsaw, Faculty of Chemistry, Biological and Chemical Research Centre, Żwirki i Wigury 101, 02089 Warsaw, Poland; ‡ University of Warsaw, Faculty of Chemistry, Pasteura 1, 02093 Warsaw, Poland; § Synchrotron Soleil, L′Orme des Merisiers, Départementale 128, 91190 Saint-Aubin, France

## Abstract

This study investigates the interfacial interactions between the selected methylxanthines, theophylline (Theo) and its derivative theophylline-7-acetic acid (TheoAcid), and model pulmonary surfactants. Chronic obstructive pulmonary disease (COPD) and asthma treatments utilizing these drugs are often limited by a narrow therapeutic window and systemic toxicity. We explore the biophysical feasibility of localized inhalation delivery by analyzing physicochemical drug effects on two-dimensional (2D) monolayers of DPPC, DPPG, and their binary mixture (8:2 molar ratio) at the air–water interface. Structural reorganization was interrogated using Brewster angle microscopy (BAM), grazing incidence X-ray diffraction (GIXD), and polarization modulation infrared reflection absorption spectroscopy (PM-IRRAS). Results demonstrate that while theophylline exerts a mild influence, theophylline-7-acetic acid significantly disrupts the organization of the lipid models. The surface properties of phospholipid membranes probed by the Langmuir technique change significantly when exposed to theophylline-7-acetic acid. GIXD analysis reveals a drug-induced transition from rectangular to hexagonal molecular packing in the DPPG monolayers. Furthermore, PM-IRRAS identifies preferential interactions with phosphate head groups, leading to changes in hydration and interfacial fluidization. The increased effect of TheoAcid compared to that of theophylline is attributed to possible electrostatic interactions, especially with negatively charged DPPG layers. These findings were also bridged to three-dimensional (3D) systems using fluorescence microscopy of giant unilamellar vesicles (GUVs), which confirmed drug-induced phase separation and morphological changes. Together, these results provide information about the physicochemical mechanisms of methylxanthine–lung surfactant interactions, offering critical insights into the stability of pulmonary interfaces under drug exposure.

## Graphical abstract

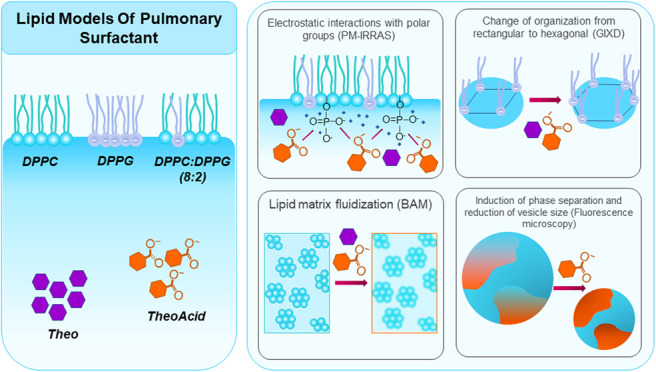

## Introduction

Pulmonary diseases constitute one of the major health issues globally. Among them, chronic obstructive pulmonary disease (COPD) is a significant cause of morbidity and mortality. It is usually caused by and associated with a prolonged exposure to air pollution and toxic substances from cigarette smoke. According to the WHO data, 64 million people have COPD and 3 million people die from COPD each year.^1^ Currently ranking as the fourth leading cause of death behind cardiovascular disease, cancer, and stroke, chronic obstructive pulmonary disease is expected to be the third leading cause of mortality in 2030.^2^ Similar to other lung diseases, COPD is a condition involving largely irreversible obstruction of the airways, and it is often characterized by alveolar wall destruction followed by the compression of airways during inspiration due to the loss of elasticity.^3,4^ It is also accompanied by airway inflammation and narrowing and excessive mucus secretion.^5^ The main means of treatment of COPD include the application of bronchodilator drugs and inhaled glucocorticosteroids and their combinations to relieve the symptoms.^6^

Methylxanthines are a class of purine-derived pharmacological agents that have been utilized for over 80 years in the management of obstructive airway diseases. Examples of methylxanthines include theophylline, its highly soluble derivative aminophylline, doxofylline, theophylline-7-acetic acid, and naturally occurring compounds such as caffeine and theobromine. Their pharmacological efficacy is primarily driven by the nonselective inhibition of phosphodiesterase enzymes. Due to their mild stimulating and bronchodilator effects, they are utilized for the treatment of asthma and chronic obstructive pulmonary disease (COPD).^7,8^ However, the therapeutic window of methylxanthines is remarkably narrow, which is why they are generally no longer considered first-line drugs.^8^ Their therapeutic and toxic ranges are closely aligned, with the toxic threshold occurring at blood concentrations of approximately 20 μg/mL.^9^ Symptoms indicating that the harmful levels of methylxanthines have been reached in the body include hand tremors, nausea, vomiting, and arrhythmia. Nevertheless, despite these safety concerns, they serve as crucial add-on therapies for patients with severe, symptomatic COPD or frequent exacerbations that remain uncontrolled by standard regimens. Additionally, one of their advantages is also their low cost. Therefore, theophylline is still one of the most widely prescribed drugs for the treatment of asthma and COPD in developing countries.

Lung surfactant (LS) plays a fundamental role in the breathing process by reducing surface tension at the air–water interface within the alveoli. The drastic changes in alveolar surface area throughout the respiratory cycle dictate that surface tension must fall below 2 mN/m at the end of expiration to prevent alveolar collapse.^10^ By doing so, pulmonary surfactant significantly reduces the work of breathing. This function is facilitated by its specific composition, which consists of 80–90% lipids, with dipalmitoylphosphatidylcholine (DPPC) serving as the predominant component.^11^ Consequently, lipid monolayers prepared at the air–water interface using the Langmuir technique have been widely employed as model systems for LS in numerous studies. DPPC monolayers, serving as the simplest model, have been extensively characterized regarding their morphology, elastic properties, and ability to respread upon expansion, which is often hindered by the formation of stable, two-dimensional crystalline domains.^12,13^ More complex and physiologically relevant models of lung surfactant incorporate mixed DPPC:POPC^14^ and DPPC:POPG^15^ monolayers. These mixed compositions better approximate native surfactants by accounting for the presence of phosphatidylglycerol (PG) lipids, the second most abundant lipid class in LS. Single-component DPPC or mixed DPPC:PG monolayers formed via the Langmuir technique have been extensively utilized as model lung surfactants by several research groups, including Perez-Gil^11,16,17^ and Sosnowski.^18,19^ These models have been used to study the interfacial interactions of various drugs administered for pulmonary diseases, including corticosteroids,^20^ anti-inflammatory^21^ or antimycobacterial^22^ agents. These studies demonstrate that contact with drugs generally leads to the fluidization of the phospholipid layers, thereby hindering the formation of liquid-condensed films. This fluidizing effect has been interpreted as a mechanism by which drugs contribute to the dynamic respreading of lung surfactant, ultimately facilitating the normal breathing process. Furthermore, it has been shown that drugs can initially accumulate within the LS lipid layer and subsequently be efficiently released during the compression–expansion dynamics characteristic of respiration.^20,22^

Despite the long-standing clinical use of methylxanthines, their direct biophysical interactions with lung surfactant (LS) remain unexplored in the current literature. In this study, we investigate the effects of the standard drug theophylline and its derivative, theophylline-7-acetic acid, on the physicochemical properties of model LS. Understanding these interfacial interactions is a critical prerequisite for the development of inhaled methylxanthine formulations. Localized pulmonary delivery represents a highly desirable alternative to oral administration as it could significantly reduce systemic toxicity and circumvent the dangerous narrow therapeutic window associated with these drugs. To precisely evaluate these interactions, our model LS utilizes dipalmitoylphosphatidylcholine (DPPC) and dipalmitoylphosphatidylglycerol (DPPG), both individually and in a mixed 8:2 molar ratio, to closely mimic the lipidic composition of a native surfactant. This particular composition involving lipids with saturated acyl chains and molar ratio reflects its ability to decrease surface tension in the alveoli and corresponds to the average content of phosphatidylcholines and phosphatidylglycerols in natural LS and therefore has been used numerous times as a model system in studies on model lung surfactants.^21,23,24^ We implemented a comprehensive, multiscale analytical approach utilizing the Langmuir technique to form planar phospholipid monolayers at the air–water interface. These experiments were complemented by Brewster angle microscopy (BAM) and grazing incidence X-ray diffraction (GIXD) to elucidate the structural reorganization of the lipid molecules at both the meso- and nanoscales. Furthermore, to bridge the 2D planar models with 3D membrane geometries, fluorescence microscopy was employed to visualize drug-induced morphological changes in spherical lipid structures (Figure 1).

**Figure 1:**
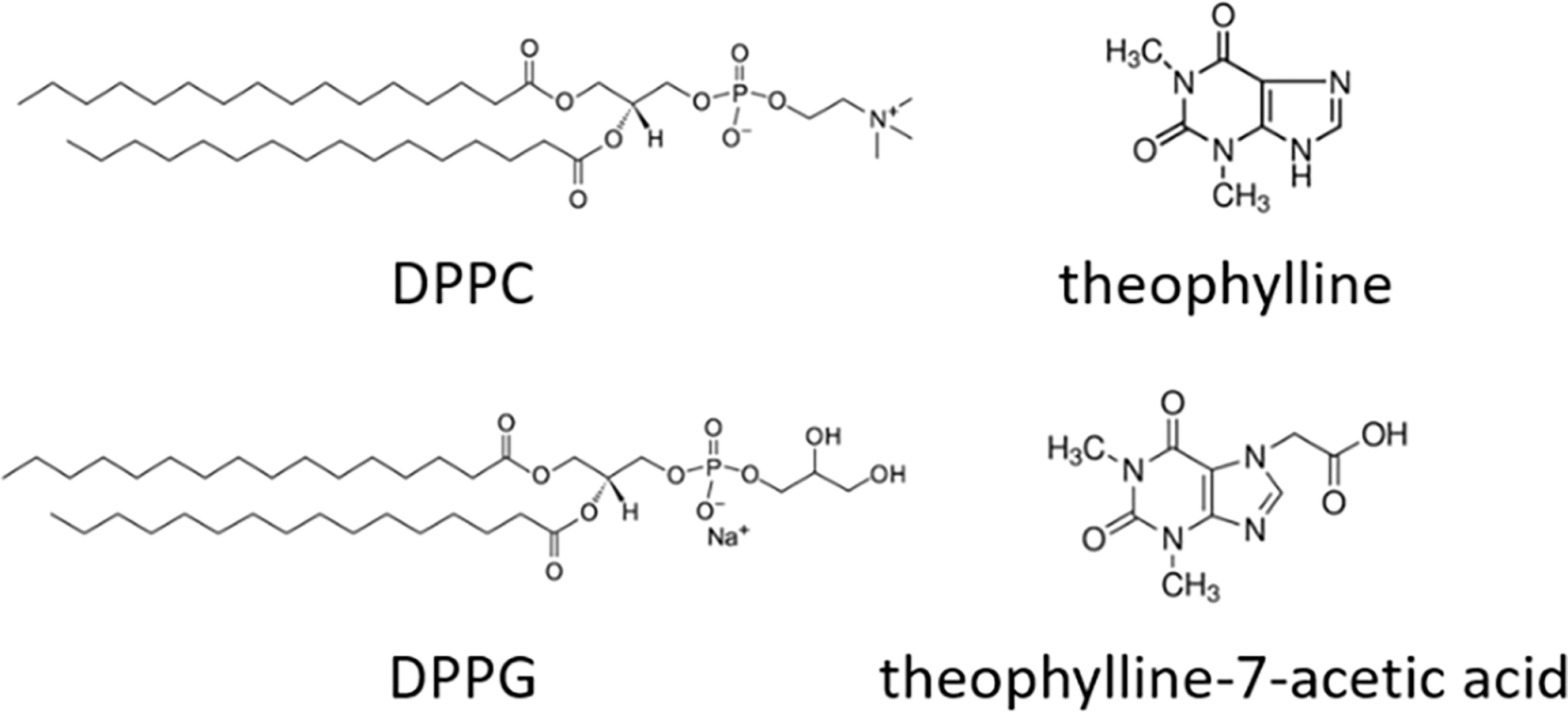
Structures of the components of lung surfactants and methylxanthines.

## Experimental Section

### Materials

1,2-Dipalmitoyl-*sn*-glycero-3-phosphocholine (DPPC) and 1,2-dipalmitoyl-*sn*-glycero-3-phospho-(1′-rac-glycerol) (sodium salt) (DPPG) were obtained from Avanti Polar Lipids (USA). Fluorescent probes: NBD-DPPC (1-palmitoyl-2-{12-[(7-nitro-2–1,3-benzoxadiazol-4-yl)­amino]­dodecanoyl}-*sn*-glycero-3-phosphocholine) and NBD-DPPG (-palmitoyl-2-{12-[(7-nitro-2–1,3-benzoxadiazol-4-yl)­amino]­dodecanoyl}-*sn*-glycero-3-phosphoglycerol) were also purchased from Avanti Polar Lipids (USA). Organic solvents: chloroform and methanol (HPLC grade), and methylxanthines: theophylline (Theo), and theophylline-7-acetic acid (TheoAcid) were purchased from Merck (Poland). Phospholipid solutions were prepared in chloroform:methanol 4:1 v/v mixture at a concentration of approximately 0.5 mg/mL. Drug solutions used as a subphase were prepared by dissolving appropriate volumes of stock solutions of theophylline (1 mg/mL in water) or theophylline-7-acetic acid (0.5 mg/mL in methanol) in Milli-Q water in order to obtain the final concentration of 10^–4^ mol/L. The subphase concentrations reflected the concentrations usually employed in the studies of drugs administered via inhalation^25,26^ as well as fall within a very narrow therapeutic range (20–100 mM), above which theophylline is toxic.^27,28^ Ultrapure Milli-Q water of resistivity 18.2 MΩ·cm was employed throughout all the experiments.

### Methods

#### Langmuir Technique

The surface pressure–area per molecule (π-*A*) isotherms were registered using a KSV NIMA Langmuir trough (total area 243 cm^2^) with two hydrophilic barriers. A Wilhelmy microbalance with a filter paper serving as a Wilhelmy plate allowed for the measurement of surface pressure with the accuracy of ±0.1 mN/m. Milli-Q water (resistivity of 18.2 MΩ·cm) or methylxanthine solutions (10^–4^ mol/L) were used as a subphase. After cleaning the subphase surface, lipid solutions were deposited at the air–water interface by a Hamilton microsyringe and left for 10 min to allow for solvent evaporation. The barrier speed was set to 10 mm/min (75 mm^2^/min). All experiments were performed at room temperature (21 °C), and each measurement was repeated at least three times to ensure the reproducibility of the results.

Isotherm data were used to calculate compression modulus (*C*_s_^–1^), which is another important parameter providing information on the elastic properties of monolayers. It is defined as the reciprocal of compressibility^29,30^

1 $$C_{s}^{-1}=-A{\left(\frac{\mathrm{d\pi}}{dA}\right)}_{T}$$

where *C*_s_^–1^ is the compression modulus, *A* is the area per molecule, and π denotes surface pressure. The maximum values of *C*_s_^–1^ give information on the phases of the monolayer, such as the gas phase (G) characterized by the values between 0 and 12.5 mN/m, liquid-expanded phase (LE) from 12.5 to 50 mN/m, liquid-condensed phase (LC) between 100 and 250 mN/m, and solid phase (S) with values above 250 mN/m.^31^ Additionally, the minima on the compression modulus vs surface pressure (*C*_s_^–1^ vs π) plots indicate the phase transitions within the monolayer.

Apart from π*-A* isotherms, the changes in the surface pressure over time were also followed. In this type of experiment, the phospholipid monolayer was first compressed to the surface pressure of 30 mN/m, which corresponds to the surface pressure, at which monolayers and bilayers are in corresponding states in terms of their organization and elastic properties.^32,33^ Then, the barriers were stopped and changes in surface pressure in time were monitored for phospholipid monolayers formed on pure water subphase, following the injection of methylxanthines. The volume of the drug solution added to the subphase was adjusted in such a way that the final concentration of 10^–4^ mol/L was obtained.

The multiple compression–expansion cycles were recorded for the monolayers within the 0–30 mN/m surface pressure range, with the upper limit corresponding to the biologically relevant surface pressure.^32^ Based on these experiments, it was possible to calculate the thermodynamic parameters of hysteresis: the free energy of hysteresis (Δ*G* ^hys^), the configurational entropy of hysteresis (Δ*S* ^hys^), and the enthalpy of hysteresis (Δ*H* ^hys^), according to the following equations^34,35^

2 $${\Delta G}_{\mathrm{comp}/\exp}=N_{A}\int_{1\;\mathrm{mN}/m}^{30\;\mathrm{mN}/m}Ad\pi$$

3 $$\Delta G^{\mathrm{hys}}=\Delta G_{\exp}-\Delta G_{\mathrm{comp}}$$

4 $${\left[\Delta S_{\pi }^{\mathrm{hys}}=R\ln\frac{A_{\exp}}{A_{\mathrm{comp}}}\right]}_{\pi }$$

5 $$\Delta S^{\mathrm{hys}}=\sum_{\pi }\Delta S_{\pi }^{\mathrm{hys}}$$

6 $$\Delta H^{\mathrm{hys}}=\Delta G^{\mathrm{hys}}+T\Delta S^{\mathrm{hys}}$$

where Δ*G*_comp/exp_ corresponds to the free energy of compression/expansion, *N*_A_ is the Avogadro number, and *R* is the gas constant. The values, which are presented in the tables, are calculated for the first compression–expansion cycle.

#### Brewster Angle Microscopy (BAM)

The morphology of phospholipid monolayers was observed by Brewster angle microscopy (BAM) using the Nanofilm Ep3 equipment with an UltraBAM objective (Accurion, Germany). The images were recorded simultaneously with the compression of the layers at the air–water interface. Each image represents a field of view of 800 μm × 430 μm and was captured with a lateral resolution of 2 μm.

#### Polarization Modulation Infrared Reflection Absorption Spectroscopy (PM-IRRAS)

Nicolet iS50 FT-IR spectrometer (Thermo Scientific, USA) equipped with ZnSe photoelastic modulator (PEM), an MCT-A liquid nitrogen-cooled detector, and a Langmuir trough (medium-size trough, KSV-Nima, Biolin Scientific, Sweden) placed in an enclosed Plexiglas cover assembly was used to collect PM-IRRAS spectra. In order to provide the stability of the measurements, the setup was placed on an optical table and was purged with dried air in order to ensure a constant vapor atmosphere. The spectra were measured using Omnic software in the wavenumber range between 4000 cm^–1^ and 800 cm^–1^ with a resolution of 8 cm^–1^ and at the incident angle of the light beam set to 70°. Each measurement consisted of 512 scans. PEM set to 1500 cm^–1^ and operating at a frequency of 50 kHz was used to ensure the constant modulation of the IR light between the *p* and *s* polarization, which allows for the simultaneous measurement of spectra for the two polarizations. The reference spectrum is defined as the sum of the intensities (*I*_s_ + *I*_p_), while their difference (*I*_s_ – *I*_p_) provides the surface-specific information. As a result, the final spectrum is defined as $S=\frac{I_{s}-I_{p}}{I_{s}+I_{p}}$ . For each measurement, the spectra were first collected for the pure subphase without phospholipid monolayer (*S*_0_) and then for the phospholipid monolayer (*S*_π_) held at a constant surface pressure of 30 mN/m. The final PM-IRRAS spectrum was defined as

7 $$\Delta S=\frac{S_{\pi }}{S_{0}}$$

A background correction procedure was employed for the final spectra. The PM-IRRAS results represent the averages of three measurements.

#### Grazing Incidence X-ray Diffraction (GIXD)

The GIXD experiments were performed at the SIRIUS beamline in SOLEIL synchrotron (Gif-sur-Yvette, France),^36^ which is equipped with the liquid surface diffractometer and the Langmuir trough (R&K GmbH Electronics, Germany) enclosed in a gastight box flushed with helium in order to reduce scattering and sample damage. The energy of the incoming X-ray beam was equal to 8 keV (λ = 1.55 Å), and the scattered signal was detected by a Pilatus3 2D pixel detector (Dectris Ltd., Switzerland) associated with a Soller collimator (JJX-ray Denmark). The spectra were obtained for phospholipid monolayers compressed to 30 mN/m (surface pressure held constant during the measurement) with the resolution of approximately 0.006 Å^–1^ by scanning the in-plane 2θ angle. Recording the vertically scattered intensity allowed us to obtain the intensity maps *I*(*Q*_*xy*_ *,Q*_*z*_), where *Q*_*xy*_ and *Q*_*z*_ are the components of the scattering vector. Conversion formulas between the scattering angles and wave-vector components were provided in our previous studies.^37–40^ The diffraction data were fitted using a linearly decreasing function to account for the background and Lorentzian functions to model the diffraction peaks. Further theoretical details concerning the GIXD technique are available in the literature.^41^

#### GUVs Preparation and Characterization

Giant unilamellar vesicles (GUVs) were obtained thanks to the electroformation method by Angelova and Dimitrov.^42^ Lipid mixture containing DPPC and DPPG (5 mg/mL concentration each) in an 8:2 molar ratio was prepared in the same way as for the Langmuir technique, but in this case doped with 1 mol % of NBD-DPPC or NBD-DPPG. The above solution was placed in a homemade chamber containing two platinum-wire electrodes.^43^ Then, the mixture solution in the chamber was allowed to evaporate under argon conditions for 1 h. The electroformation was carried out in a 200 mmol/L sucrose solution (in phosphate-buffered saline, pH = 7.4) using a low-frequency alternating field (10 Hz and amplitude of 1.0 V, 90 min).^44^ Millicell EZ SLIDE 8-well glass (Merck KGaA, Darmstadt, Germany) was used to observe GUVs. For this, 50 μL of GUVs in sucrose solution was placed in a well containing 200 μL of an isomolar glucose solution (200 mmol/L, in phosphate-buffered saline, pH = 7.4).^45^ Methylxanthines (theophylline and theophylline-7-acetic acid) prepared as described above were added into single wells to a final concentration of 10^–4^ mol/L. The effect of drugs on GUVs was monitored for 15 min in a fluorescence microscope. The experiments were carried out at room temperature (21 °C). Images were captured using the JENOPTIK GRYPHAX software with a Nikon Eclipse Ni–U microscope (Tokyo, Japan).

## Results and Discussion

### Interactions of Methylxanthines with DPPC Single-Component Langmuir Monolayers at the Air–Water Interface

Interactions of methylxanthines with single components of lung surfactants were verified using Langmuir films at the air–water interface. Since phosphatidylcholines, and especially DPPC, are the main components of a lung surfactant,^11,16,46,47^ the monolayers of DPPC were first formed at the air–water interface to observe the typical π-*A* isotherm of this phospholipid.^48,49^ Three phases are classically detected on the isotherm. Upon compression, the gas (G) to liquid expanded (LE) phase transition starting at zero surface pressure is observed, followed by a surface pressure plateau corresponding to the LE-LC (liquid expanded–liquid condensed) transition. Next, the LC phase and the collapse are measured. The presence of the drugs in the subphase changes the monolayer organization and surface properties, which is evidenced by an altered shape of the isotherms (Figure 2). However, the effect strongly depends on the type of methylxanthine used. Theophylline leads to a shift of the plateau region of the DPPC isotherm toward slightly higher surface pressure and its elongation with the concomitant changes in the slope of the isotherm following further compression of the monolayer. The other investigated methylxanthine, theophylline-7-acetic acid, leads to significant changes in the shape of the isotherms—the characteristic plateau region shifts toward lower surface pressure and is not that well-developed. Additionally, the monolayer collapses at much lower surface pressure (Table S1), which suggests that TheoAcid reduces the stability of DPPC monolayers.

**Figure 2:**
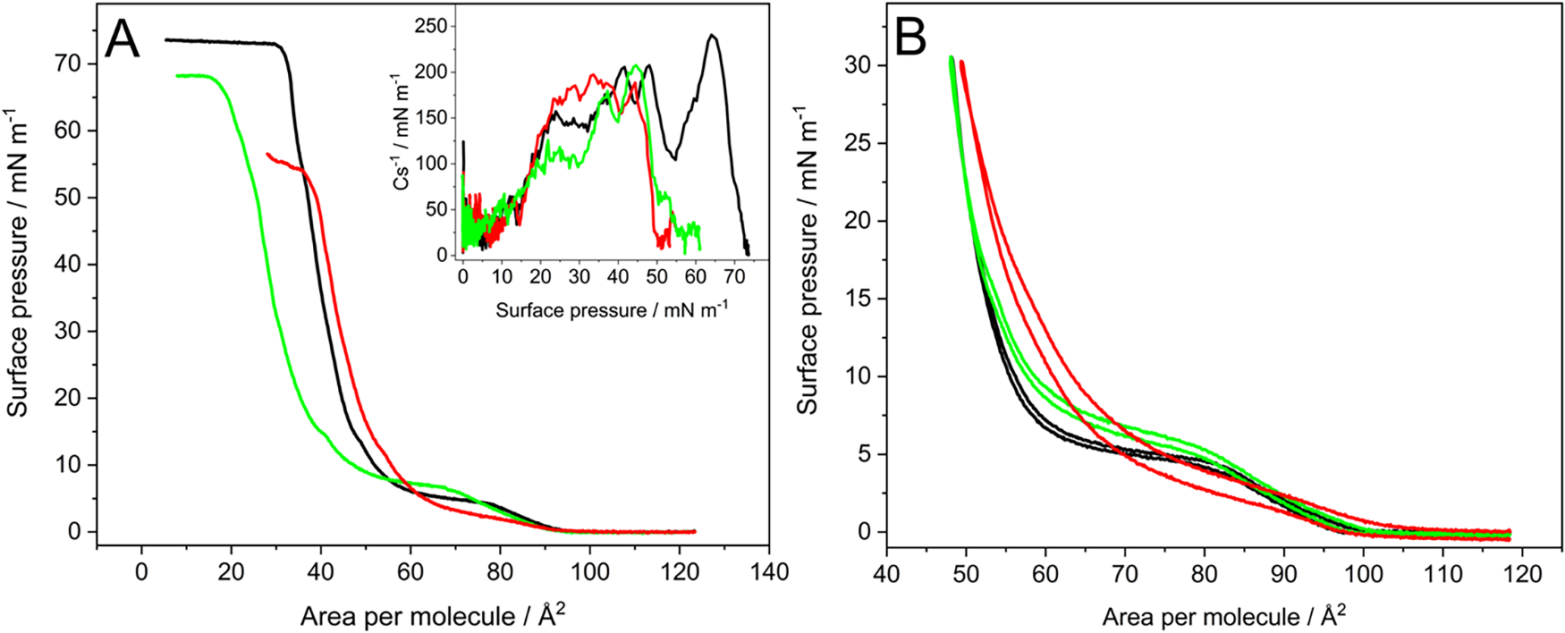
(A) Surface pressure–area per molecule (π-*A*) isotherms; (B) compression–expansion cycles of DPPC monolayers on a pure water subphase (black) and water subphase with 10^–4^ mol/L theophylline (green), theophylline-7-acetic acid (red). Inset: compression modulus versus surface pressure (*C*_s_^1–^- π) plots.

A more detailed explanation of the effect of methylxanthines on the reversibility of the DPPC monolayers can be obtained based on the multiple compression–expansion cycles (Figure 2B). Such experiments performed in laboratory conditions may mimic the breathing process taking place in the alveoli. The thermodynamic functions of hysteresis calculated based on the compression–expansion cycles (Table S2) provide information on the processes associated with the possible formation of aggregates due to the adhesion forces and the viscosity of the layers and may provide indirect information on the effectiveness of the breathing process.^34,35^ For ideally behaving layers, the thermodynamic parameters should be equal to 0 without any hysteresis. However, even for DPPC monolayers formed on pure water subphase, the slightly negative values are observed, and these parameters are not significantly altered in the presence of theophylline, although the observed differences are statistically meaningful (Table S2). More pronounced changes are visible when the DPPC monolayer is compressed and expanded on the subphase containing theophylline-7-acetic acid. More negative value of the free energy of hysteresis (Δ*G* ^hys^) suggests the formation of aggregates or assemblies as a result of cohesive intra- and intermolecular forces within the monolayer, while the more negative value of *T*Δ*S* ^hys^ proves the presence of entropically unfavorable interactions. The much more negative value of Δ*H* ^hys^ points to the enthalpically favorable interactions (Table S2). These statistically significant changes in the thermodynamic functions of hysteresis due to the presence of TheoAcid suggest that this methylxanthine may affect the surface properties connected with the reversibility of the DPPC component of a lung surfactant.

More in-depth information on the organization of the phospholipid molecules in the monolayer in the presence and absence of methylxanthines can be obtained from the analysis of the changes in the compression modulus, which is also affected. Interestingly, both medicines reduce the maximum of *C*_s_^–1^ to a value of approximately 190–200 mN/m compared to 225 mN/m for pure DPPC monolayer, which is not significant but still points to its slight fluidization (inset in Figure 2 and Table S1). This observation is also supported by the BAM images recorded for DPPC monolayers formed on different subphases (Figure 3). The typical DPPC domains can be observed for monolayers formed on pure water subphase.^50–53^ Theophylline induces a decrease in the size of the domains, although the shape is not altered. The effect of the theophylline-7-acetic acid is significantly different and prevents regular domain formation and growth upon the compression of the layer. Only a few small bright spots can be observed at low surface pressure of approximately 2–5 mN/m, which do not grow to form typical DPPC domains but rather remain very small with the compression of the layer. Eventually, a uniform monolayer is formed, which suggests that the small-dotted domains merge. It suggests that even theophylline-7-acetic acid, although leads to some fluidization of the DPPC monolayer, is not able to completely change its organization. It is further supported by the results of grazing incidence X-ray diffraction data obtained for DPPC monolayer compressed to 30 mN/m in the absence and presence of TheoAcid (Figure 4). The typical diffraction pattern of DPPC^54^ remains unchanged even when the methylxanthine is present in the subphase, corresponding to a rectangular unit cell with a NN tilt of the alkyl chains. However, some of the characteristic GIXD parameters are affected (Table S3) despite the fact that the rectangular structure with NN tilted chains remains. Especially, the tilt angle (τ) and the area of a unit cell (*A*_uc_) increase slightly, which suggests that the unit cell parameters are modulated by the presence of methylxanthine. Interestingly, the increase of the unit cell area is in agreement with the increase of macroscopic area measured on the isotherms. Additionally, the in-plane coherence length (*L*_*XY*_) related to the range of 2D crystallinity is significantly reduced, which further supports the observations on the fluidizing and disorganizing effect of TheoAcid on DPPC monolayers as observed in the mesoscale based on the compression modulus values and Brewster angle microscopy images.

**Figure 3:**
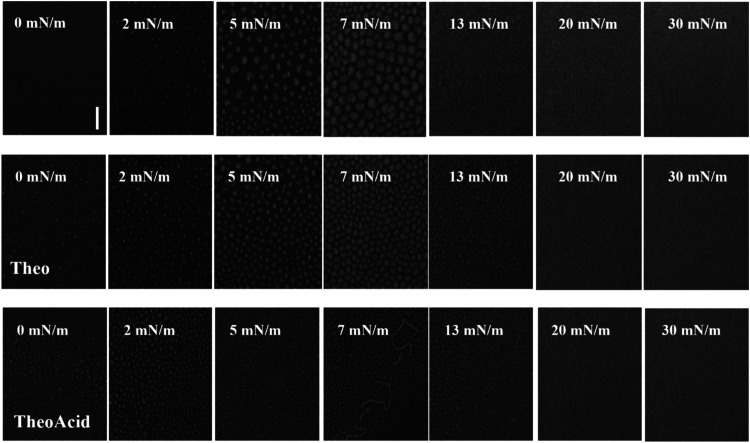
BAM images obtained at selected surface pressures for the DPPC monolayers on a pure water subphase and water subphase with 10^–4^ mol/L theophylline and theophylline-7-acetic acid. The scale bar is 100 μm.

**Figure 4:**
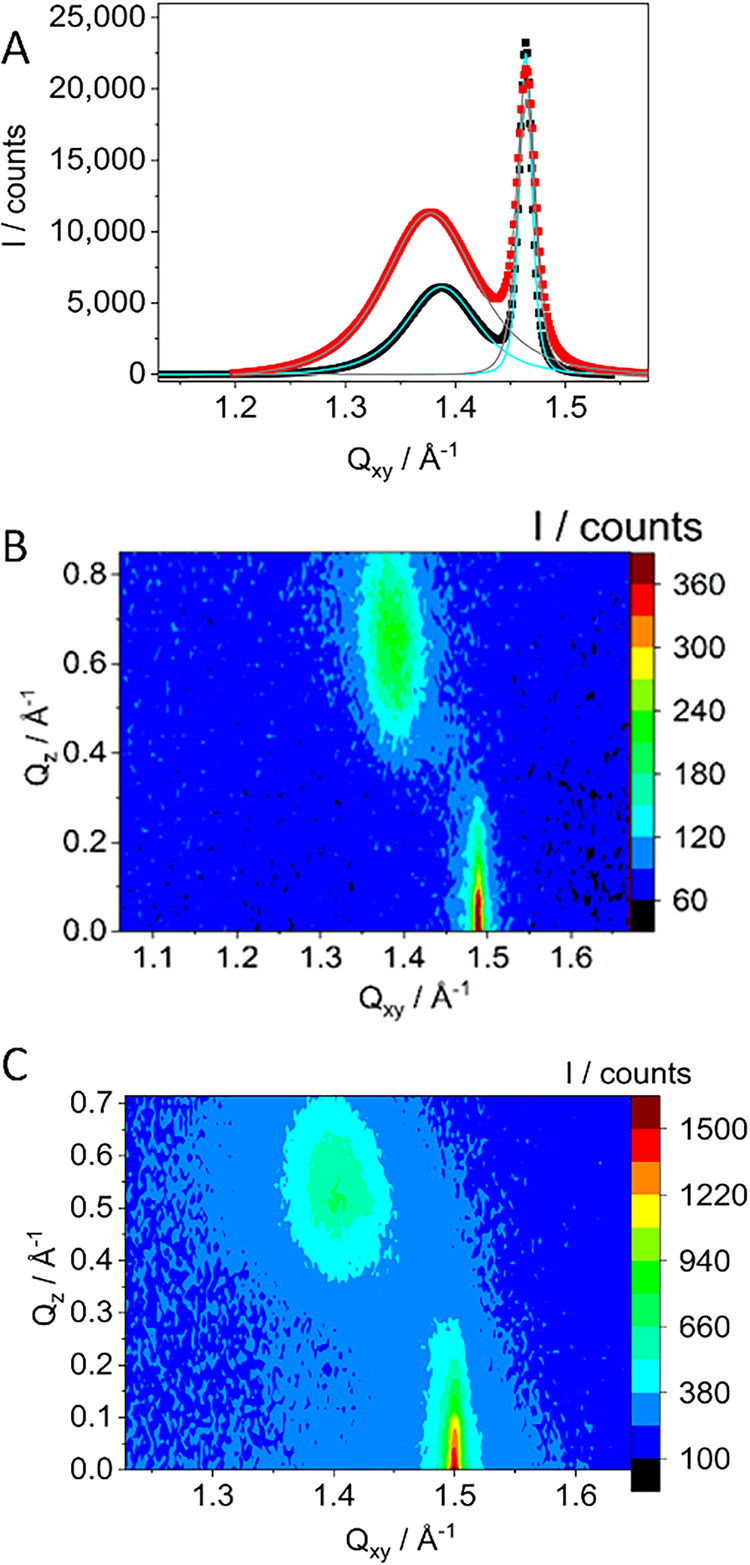
(A) GIXD *Q*_*z*_-integrated Bragg peak profiles of DPPC monolayers compressed to 30 mN/m on pure water subphase (black) and water subphase containing 10^–4^ mol/L theophylline-7-acetic acid (red). The corresponding *Q*_*Z*_ *-Q*_*XY*_ intensity maps for DPPC Langmuir monolayers on (B) pure water subphase and (C) water subphase with 10^–4^ mol/L theophylline-7-acetic acid. Solid black and red points are experimental data; gray and blue lines are Lorentz curve fits.

### Interactions of Methylxanthines with DPPG Single-Component Langmuir Monolayers at the Air–Water Interface

Another important component of a lung surfactant includes a representative of phospatidylglycerols (PG), namely 1,2-dipalmitoyl-*sn*-glycero-3-phospho-(1′-rac-glycerol) (DPPG). This phospholipid differs in its structure from DPPC (Figure 1) and thus also in its surface properties. The main difference lies in the composition of the polar head, resulting in its negative charge. DPPG on pure water surface forms well-organized, closely packed monolayers of a solid character as evidenced by relatively high values of compression modulus (Figure 5A). Such surface behavior is consistent with literature reports.^55,56^ Interestingly, the presence of theophylline in the subphase does not impose significant changes in the position and shape of the π-*A* isotherms, suggesting almost no influence on the surface properties of DPPG monolayers (Figure 5A and Table S1). Consequently, the compression–expansion cycles (Figure 5B) as well as the thermodynamic functions of hysteresis (Table S2) are not affected. Conversely, the effect of theophylline-7-acetic acid is much more pronounced, since the presence of this drug in the subphase changes the shape of the isotherm. A kink or a pseudo-surface pressure plateau on the isotherm starts to be visible, followed by the formation of a more organized phase. However, a much lower maximum value of compression modulus is observed (inset in Figure 5A and Table S1). Additionally, the maximum of *C*_s_^–1^ versus surface pressure plot is located at lower values of surface pressure in the presence of theophylline-7-acetic acid compared to the DPPG monolayer formed on pure water subphase. This more pronounced effect of TheoAcid is also reflected in the hysteresis and more negative values of the thermodynamic functions of hysteresis compared to pure water subphase (Figure 5B and Table S2). It suggests that, similarly to DPPC monolayers, the irreversible, entropically unfavorable aggregates are formed due to the enthalpically favorable interactions of cohesive intra- and intermolecular nature.^34,35^ These observed changes in the surface properties of DPPG monolayers exposed to TheoAcid may be explained by the presence of electrostatic interactions between the negatively charged polar heads of DPPG and theophylline-7-acetic acid, which is in the ionized form at neutral pH. These repulsions may disrupt the well-packed organization of DPPG monolayers at the initial stages of compression in a much more distinct way than the interactions between neutral theophylline and DPPG.

**Figure 5:**
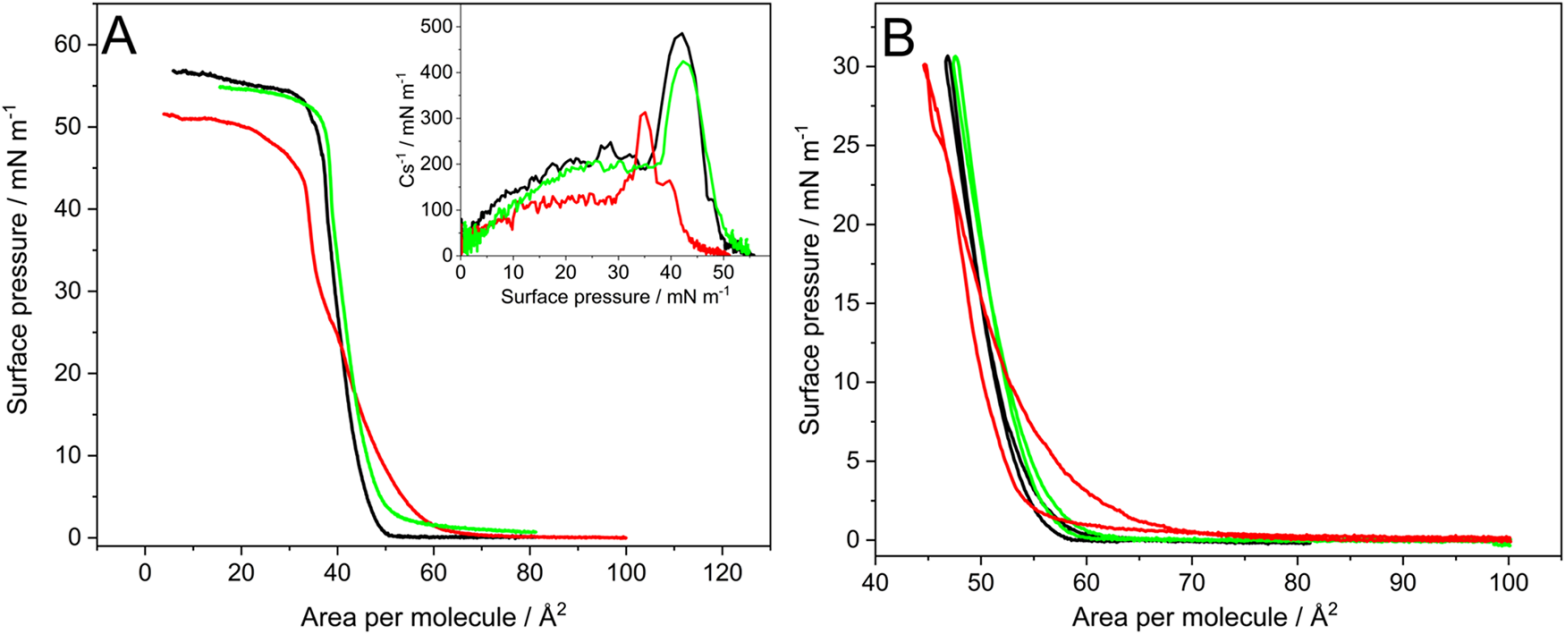
(A) Surface pressure–area per molecule (π-*A*) isotherms and (B) compression–expansion cycles of DPPG monolayers on a pure water subphase (black) and water subphase with 10^–4^ mol/L theophylline (green), theophylline-7-acetic acid (red). Inset: compression modulus versus surface pressure (*C*_s_^1–^- π) plots.

The observations of the effect of methylxanthines on the DPPG monolayers based on Langmuir monolayer experiments are also confirmed by BAM imaging. DPPG forms very well-organized, tightly packed monolayers without any distinct phase transitions.^55,57^ As a result, no domain formation is visible, and even at relatively low surface pressures, a well-packed, dense layer is observed through BAM (Figure 6, upper row). Consequently, theophylline does not induce any significant changes in the morphology of a DPPG monolayer. However, theophylline-7-acetic acid seems to induce faster organization of the layer, resulting in a much more organized, dense monolayer obtained even at very low surface pressures of 2–5 mN/m (Figure 6, lower row). At 30 mN/m, corresponding to the best organization of the layer exhibited by the maximum value of compression modulus, brighter patches were observed. Interestingly, at this surface pressure, the isotherm obtained in the presence of theophylline-7-acetic acid intersects that of DPPG on pure water, which may even suggest some expulsion of the material from the interface, probably due to the aforementioned electrostatic repulsions. Similar observations have been made before for the anticancer drugs doxorubicin and idarubicin interacting electrostatically with negatively charged DMPS monolayers.^58^ The expulsion of the drugs from the phospholipid monolayer at higher surface pressures exhibited by the similar intersections of the isotherms was also confirmed by neutron reflectivity studies.

**Figure 6:**
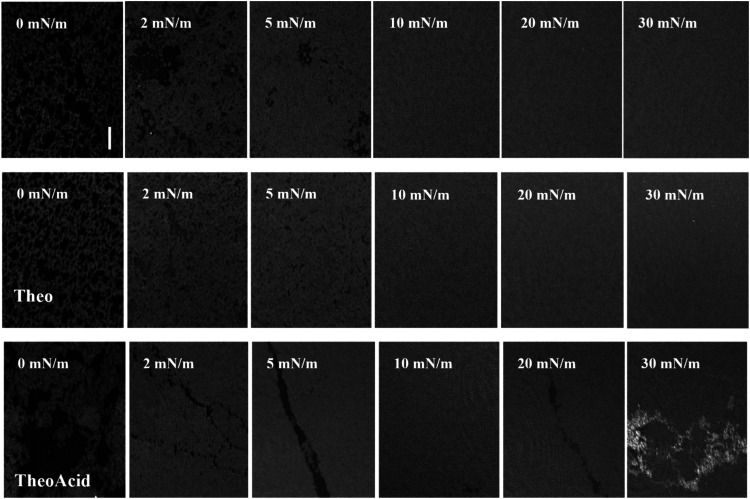
BAM images obtained at selected surface pressures for the DPPG monolayers on a pure water subphase and water subphase with 10^–4^ mol/L theophylline and theophylline-7-acetic acid. The scale bar is 100 μm.

Further information on the changes in the organization of the DPPG monolayer in the presence of TheoAcid is available from GIXD results (Figure 7). DPPG monolayer formed on pure water subphase and compressed to 30 mN/m shows a typical diffraction pattern with two peaks pointing to the rectangular organization of the molecules at the air–water interface with NN tilt of the alkyl chains (Figure 7 and Table S2).^55,57,59^ However, the presence of the selected methylxanthine in the subphase leads to significant changes in the diffraction pattern—only one in-plane peak becomes visible, which suggests the hexagonal organization of DPPG molecules at the interface with upright orientation. The peak position of 1.50 Å^–1^ corresponds to a lattice spacing of 4.83 Å, which implies a more condensed configuration of the alkyl chains. The decreased area of the unit cell also compares with the area per molecule on the isotherm that is smaller in the presence of TheoAcid. This observation is also consistent with the values of compression modulus—in the presence of TheoAcid, the maximum value of *C*_s_^–1^ is observed at 30 mN/m and is higher than the value of *C*_s_^–1^ recorded for DPPG monolayer on pure water subphase at this surface pressure (inset in Figure 5A). Therefore, it may be supposed that theophylline-7-acetic acid induces more packed organization of DPPG molecules at this surface pressure, which are organized in a tighter monolayer at the air–water interface with the hydrocarbon chains oriented vertically. This change in the organization of the monolayer is caused by TheoAcid, which is expulsed from the layer at the interface. However, after the expulsion, the drug probably stays in the vicinity of the interface and the polar heads of DPPG, changing the interactions between head groups by, e.g., interfering in the formation of H-bonding and as a result allowing the van der Waals interaction between alkyl chains to impose the more compact hexagonal organization of the layer. Such an assumption seems to be in accordance with the observed intersection of the isotherms (Figure 5A) and the brighter areas on the BAM images, suggesting the formation of more condensed, better-organized regions (Figure 6). The intersection of the isotherm recorded in the presence of TheoAcid with the isotherm obtained for DPPG on pure water subphase may also be interpreted in such a way that during the compression of the layer, not only the drug is removed from the interface but also there is some lipid loss resulting in the observed intersection of the isotherms. A similar situation was observed before in the case of the electrostatic interactions of anthracyclines^58^ or cubosomes^60^ interacting with negatively charged DMPS monolayers. In the former case, the neutron reflectivity studies revealed that partially expulsed doxorubicin was accumulating and forming a sublayer beneath the lipid head groups. Therefore, in order to probe the interactions of theophylline-7-acetic acid with polar heads of DPPG, polarization modulation infrared reflection absorption spectroscopy at the air–water interface was employed (Figure 8).

**Figure 7:**
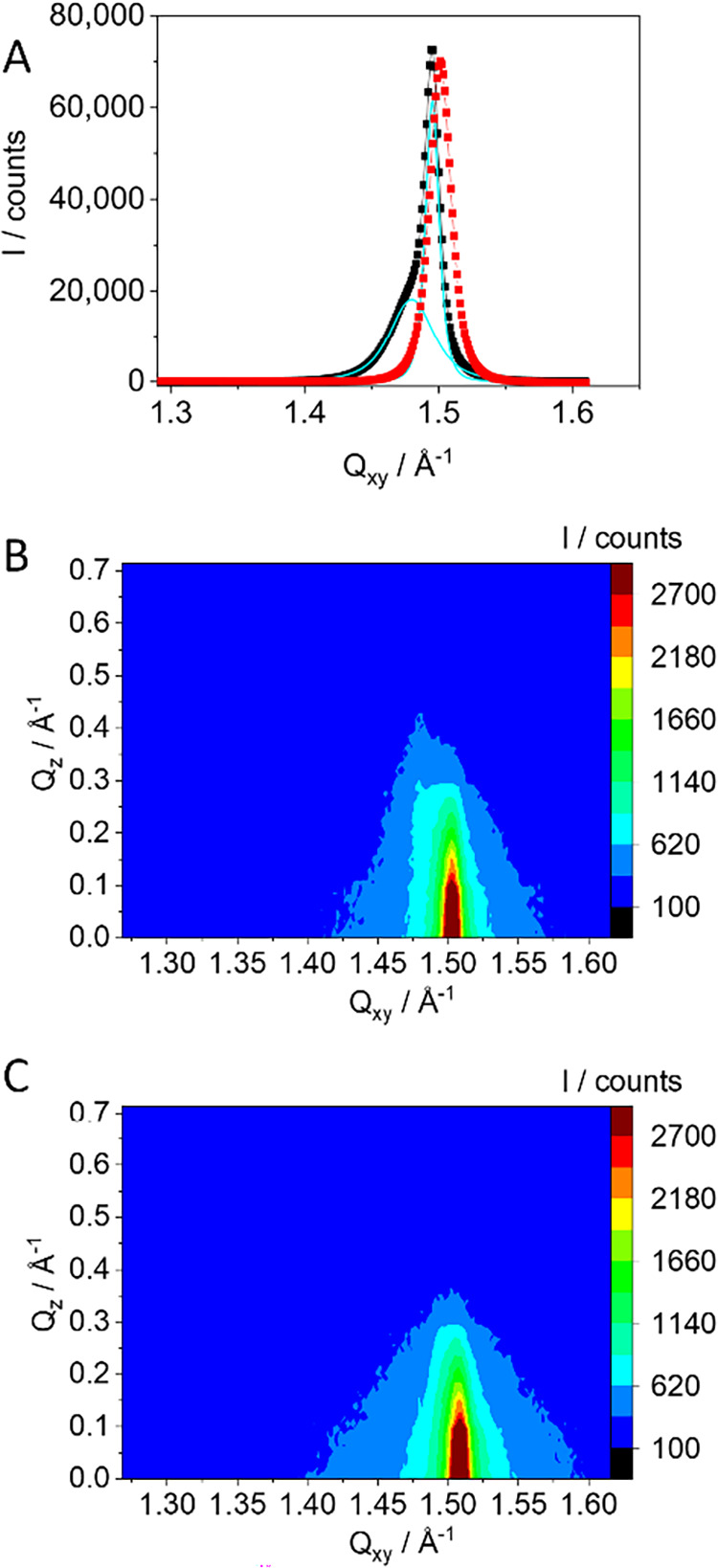
(A) GIXD *Q*_*z*_-integrated Bragg peak profiles of DPPG monolayers compressed to 30 mN/m on pure water subphase (black) and water subphase containing 10^–4^ mol/L theophylline-7-acetic acid (red). The corresponding *Q*_*Z*_ *-Q*_*XY*_ intensity maps for DPPG Langmuir monolayers on (B) pure water subphase and (C) water subphase with 10^–4^ mol/L theophylline-7-acetic acid. Solid black and red points are experimental data, gray and blue lines are Lorentz curve fits.

**Figure 8:**
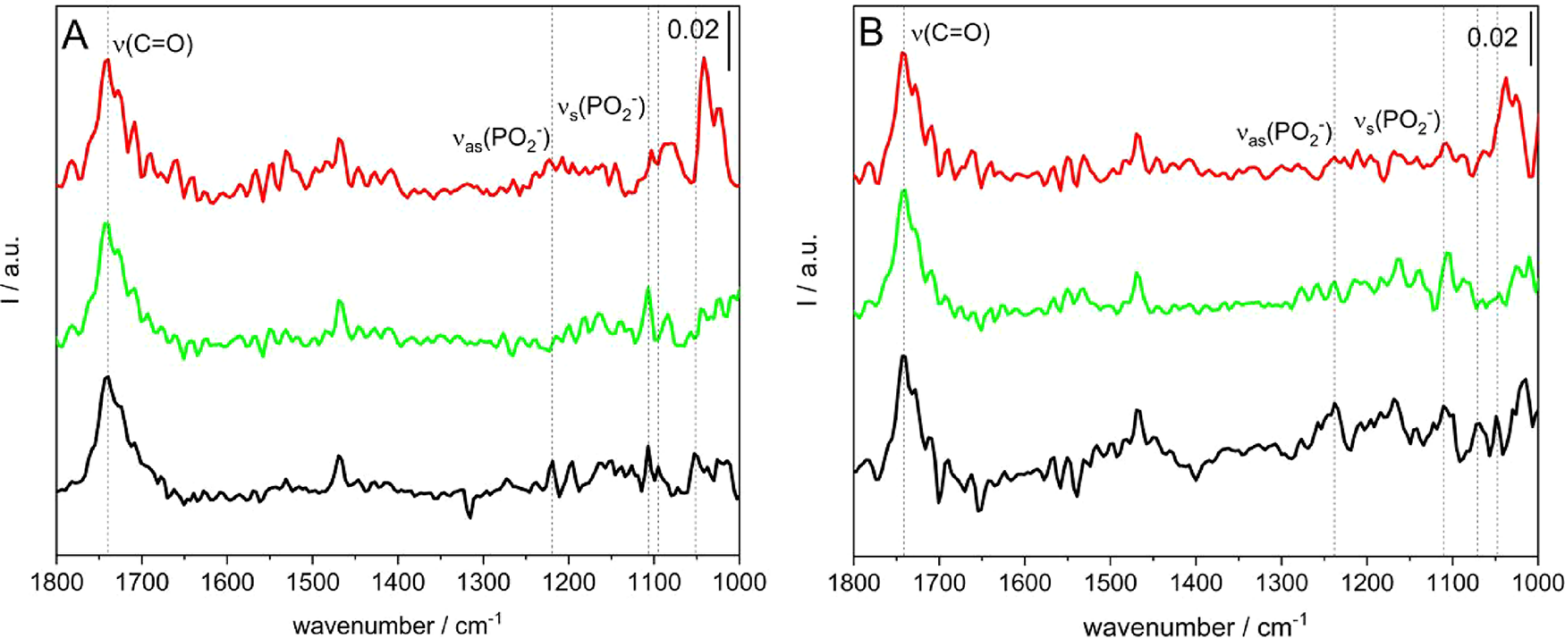
PM-IRRAS spectra of DPPG monolayers compressed to (A) 10 mN/m and (B) 30 mN/m on pure water subphase (black) and a water subphase containing 10^–4^ mol/L solution of theophylline (green) and theophylline-7-acetic acid (red) in the ∼1800 cm^–1^ to ∼1000 cm^–1^ spectral region (polar headgroup and carbonyl ester).

The spectra in the ∼1800 cm^–1^ to ∼1000 cm^–1^ spectral region covering the polar headgroup and carbonyl ester bands recorded for the monolayers compressed to two different surface pressures: 10 and 30 mN/m revealed that at lower surface pressure the bands corresponding to the carbonyl ester group show a peak located at 1739 cm^–1^ indicating its dehydration typical for DPPG layers (Figure 8A and Table S3).^61,62^ Interestingly, the location of this band is not affected by the presence of methylxanthines. There is also a low-frequency shoulder around 1724 cm^–1^, which could be attributed to the hydrogen bonds between the ester groups and the glycerol O–H groups of adjacent DPPG molecules, which is more likely to occur at low surface pressures due to the higher disorder in the DPPG film.^62^ In the case of the phosphate group vibrations at lower surface pressure, the position of the ν_as_(PO_2_^–^) band at 1218 cm^–1^ suggests the hydration of the phosphate group of the phospholipids.^63,64^ This band is also accompanied by the symmetric PO_2_^–^ vibration observed at approximately 1090 cm^–1^ and other bands located at approximately 1107 and 1050 cm^–1^, which correspond to O–P–O stretching vibrations as well as other stretching vibrations, such as, e.g., P–O­[C]. Interestingly, these bands are slightly shifted to lower wavenumbers in the presence of theophylline and even more shifted in the presence of theophylline-7-acetic acid. It suggests moderate interactions of methylxanthines with this part of the polar head, which are definitely stronger for TheoAcid, inducing the increased hydration of phosphate groups.^64,65^

Compression of the DPPG monolayer to higher surface pressure, inducing more compact orientation of the lipids at the air–water interface, results in the shift of the ν_as_(PO_2_^–^) band to higher wavenumbers, suggesting the increased dehydration of this part of the molecule, which is reasonable, since DPPG adopts a more upright position and therefore the carbonyl group becomes more dehydrated. Additionally, the presence of methylxanthines enhances this orientational change slightly by moving the phosphate band to even higher wavenumbers (Table S3). Consequently, the phosphate asymmetric band now at 1238 cm^–1^ is also shifted to higher wavenumbers, showing more dehydration^63,64^ but its position is not further affected by methylxanthines. It would suggest that at the higher surface pressure and better organization of the DPPG monolayer the drugs are not able to penetrate the monolayer more deeply to interact with carbonyl ester groups. The other bands seem to be only slightly affected by methylxanthines. Therefore, the observed trends might be interpreted in the following way: the methylxanthine interactions at lower surface pressure are stronger for the acidic form of theophylline due to the possible electrostatic repulsions affecting the polar heads of DPPG. At this stage of the organization of the layer, the drugs may affect polar heads through electrostatic interactions as well as possible hydrophobic interactions. At higher surface pressures, the differences between the two drugs are less pronounced, which might suggest that indeed the theophylline-7-acetic acid is expulsed from the interfacial layer but stays within the proximity of the polar heads, affecting the organization of the DPPG layer, as shown by the results of Langmuir and GIXD studies. The expulsion of the drug from the monolayer at higher surface pressures is also further indirectly confirmed by the positions of the CH vibrations of DPPG (Figure S1). The ν_as_(CH_2_) and ν_s_(CH_2_) bands are located at 2919 and 2850 cm^–1^, respectively. It indicates a fully stretched, *all-trans* conformation of acyl chains, which is typical for this phospholipid.^66^ In the presence of methylxanthines, the position and width of the CH bands remain similar, with the only exception of DPPG monolayer compressed to 30 mN/m on the subphase containing theophylline-7-acetic acid, when the ν_as_(CH_2_) band shifts slightly to even lower wavenumbers (2915 cm^–1^). It may suggest increased ordering of the acyl chains (decrease in acyl chain flexibility), probably due to the electrostatic interactions of theophylline-7-acetic acid remaining in the proximity of the layer and possibly interacting weakly with polar heads by interfering with the H-bonding between DPPG molecules. This may impose changes in the organization of DPPG molecules as also evidenced by the changes in the organization of the monolayer observed by GIXD (Figure 7). Similar mechanisms of interaction were also observed for DPPG layers exposed to agomelatine, an antidepressant drug.^67^

### Interactions of Methylxanthines with Binary Langmuir Monolayers at the Air–Water Interface

In order to further probe the interactions between methylxanthines and pulmonary surfactants, a more precise model consisting of the mixed DPPC:DPPG (8:2 molar ratio) monolayers was employed. The composition of the mixed layer reflects more accurately the real lipid composition of pulmonary surfactant.^11,16,46,47,68^ The mixture of lipids forms a stable monolayer at the air–water interface with the compression modulus reaching the upper limit of the LC phase (Figure 9A). The effect of methylxanthines on the surface properties of the mixed layer reflects the above-described effect on the monolayers of the single components. Since DPPC is the major lipid in the mixture, the influence of drugs is mild consistently with the results shown for DPPC monolayers (Section 3.1). While the changes in the shape of the isotherm and the maximum value of compression modulus are limited, the biggest effect concerns the decrease in the surface pressure of the collapse, suggesting the decreased stability of the layer, which is observed for both theophylline and theophylline-7-acetic acid.

**Figure 9:**
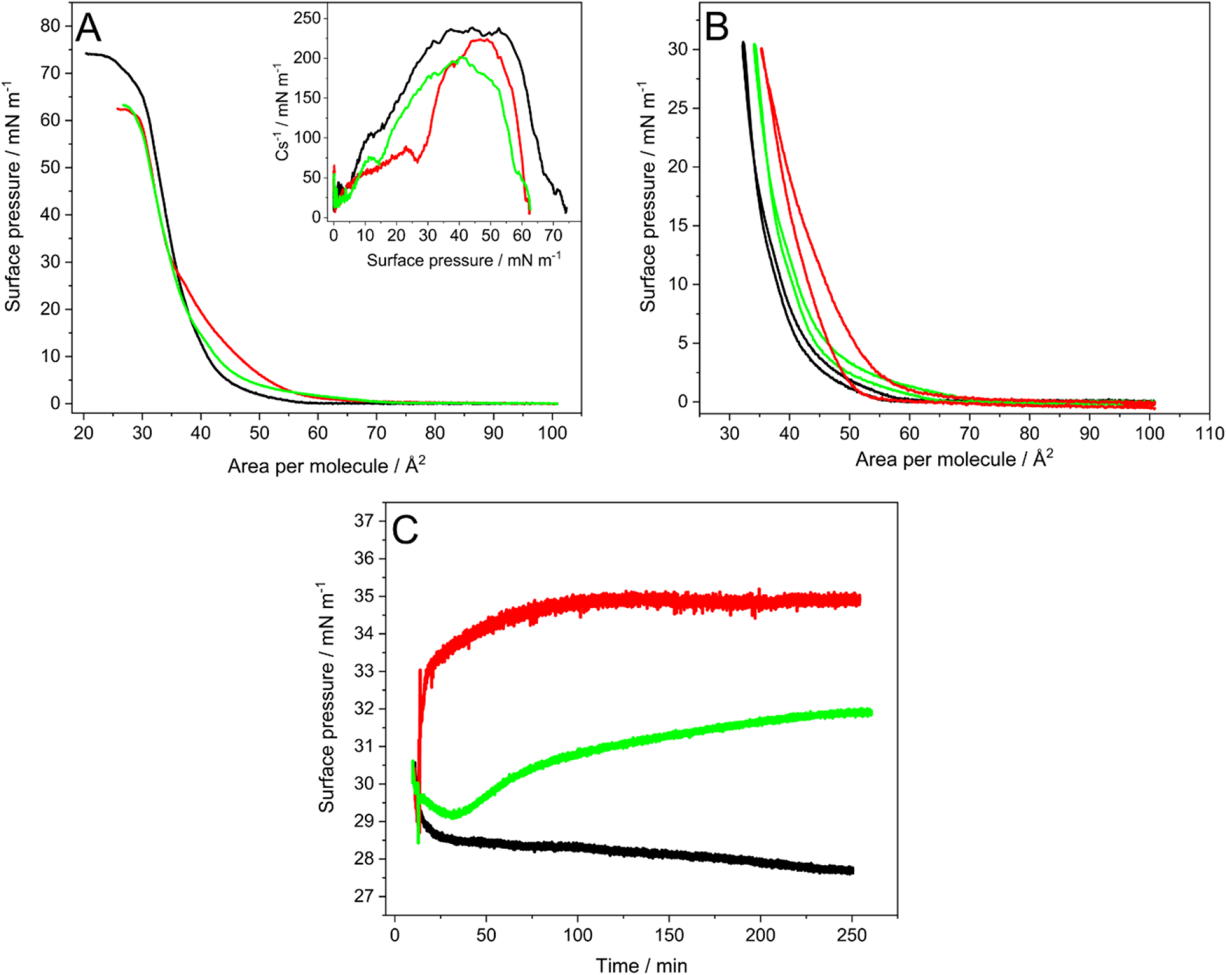
(A) Surface pressure–area per molecule (π-*A*) isotherms; (B) compression–expansion cycles; and (C) changes of the surface pressure in time of DPPC:DPPG 8:2 monolayers on a pure water subphase (black) and water subphase with 10^–4^ mol/L theophylline (green), theophylline-7-acetic acid (red). Inset: compression modulus versus surface pressure (*C*_s_^1–^- π) plots.

However, despite the limited changes in π-*A* isotherms, the reversibility of the model system is much more affected, especially in the presence of theophylline-7-acetic acid (Figure 9B). Based on the consecutive compression–expansion cycles, the thermodynamic functions of hysteresis were again calculated (Table S2). It was noticed that the free energy of hysteresis (Δ*G* ^hys^), the configurational entropy of hysteresis (*T*Δ*S* ^hys^), and the enthalpy of hysteresis (Δ*H* ^hys^) become much more negative, which again points to the formation of irreversible, entropically unfavorable aggregates as a result of the enthalpically favorable, cohesive interactions existing within the monolayer.^34,35^ Interestingly, the aforementioned values of thermodynamic functions of hysteresis are the most negative for the mixed DPPC:DPPG monolayers formed on the subphase containing theophylline-7-acetic acid, which suggests that the effect of this methylxanthine is the most pronounced for the mixed layer compared to single-component monolayers (Table S2). It also shows that the interactions within the lipids themselves in the mixed layer may also, to some point, determine the observed interactions with drugs.

Another type of experiment was also performed to probe the possibility of methylxanthines penetrating the lipid layers over time. The DPPC:DPPG monolayers were first compressed to a selected, biologically important target surface pressure of 30 mN/m,^32,33^ then the drug solutions were injected into the subphase to obtain the desired final concentration, and the changes in time were monitored (Figure 9C). The mixed layer itself is quite stable, and the surface pressure decreases only slightly during 4 h. However, the addition of theophylline at first slows down the decrease in the surface pressure in time and then leads to an increase above the starting value after 4 h. The effect of theophylline-7-acetic acid is much more pronounced and results in an immediate increase in surface pressure until the value of 35 mN/m, which remains stable throughout the measurement period. These results prove that methylxanthines, especially theophylline-7-acetic acid, may effectively interact with already organized phospholipid monolayer. However, based on the previous results, it may be supposed that the interactions are limited to polar head groups of the layer, probably without a deep penetration into the already compressed layer.

Additional information about the interactions between the drugs and the model mixed membranes was also provided by BAM images (Figure 10). Interestingly, the mixed layer formed on pure water subphase shows the formation of round, small domains at lower surface pressures, which then converge and form a more uniform but yet not completely homogeneous monolayer even at the higher surface pressures. In this way, the morphology of the mixed layer represents to some point the features of both components: DPPC and DPPG. However, the addition of theophylline results in more developed, round-shaped domains, resembling the morphology of pure DPPC monolayers exposed to theophylline. In contrast, the DPPC:DPPG monolayers exposed to theophylline-7-acetic acid produce images resembling the pure DPPG monolayers in the presence of this drug. Therefore, it shows how different the interactions of the two representatives of methylxanthines are on the mesoscale with respect to the morphology of the layer.

**Figure 10:**
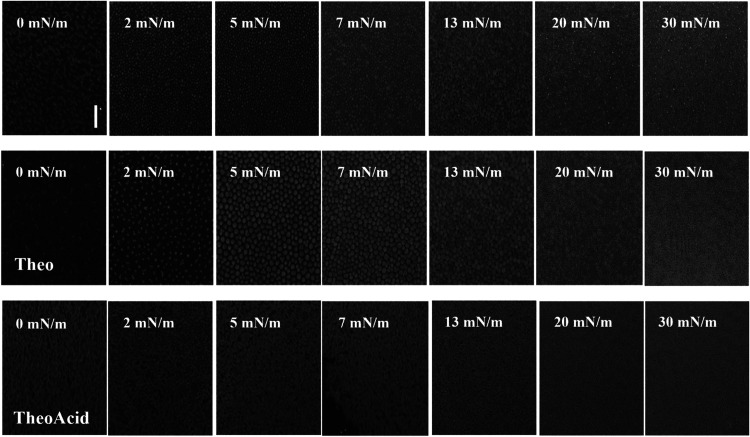
BAM images obtained at selected surface pressures for the mixed DPPC:DPPG 8:2 monolayers on a pure water subphase and water subphase with methylxanthines. The scale bar is 100 μm.

### Interactions of Methylxanthines with GUVs

In order to get additional information on the interactions of methylxanthines with lipid layers including the formation of vesicles during the breathing process,^11,16,47^ the giant unilamellar vesicles (GUVs) were also visualized to assess the impact of these drugs on the spherical lipid structures formed by electroformation (Figure 11).^42,69^ Measurements were carried out over 15 min, as untreated vesicles lost sufficient phase contrast after that time. Starting with nondrug-exposed vesicles, shapes of similar size were observed regardless of the labeled lipid (DPPC* or DPPG*), while increased phase separation was observed when lipid mixtures were doped with NBD-DPPG. Following Steinkühler et al.,^69^ the absence of visible fluctuations in the lipid membrane may indicate a high surface tension of the vesicles. In contrast, GUVs treated with theophylline showed significant drug-lipid membrane interactions. The fluorescence intensity enhanced by the presence of theophylline was more stable over time in the case of DPPC doping. However, it should be noted that a higher intensity during the theophylline action appears when the labeling was done by NBD-DPPG, except that in this case, this intensity disappears after about 10 min. The loss of lipid asymmetry may result from flip-flop movements in the membrane bilayer over time.^70^ In both cases, the presence of theophylline causes the appearance of phase separation and/or asymmetry of the lipid bilayer, which may be the result of preferential interaction of the drug with a particular model membrane component. An interesting phenomenon was the relative reduction in the size of GUVs under the influence of theophylline-7-acetic acid, which was even more pronounced in the case of NBD-DPPC doping. Perhaps this is due to the specific interactions of DPPG-theophylline acid, compared with the Langmuir monolayer studies (Figure 5), where we observed a significant reduction in the surface area per molecule after exceeding the physiological pressure. On the other hand, in the presence of theophylline-7-acetic acid, greater aggregation of lipid vesicles labeled with NBD-DPPG was observed, as well as a strong intensity and phase contrast throughout the experiment.

**Figure 11:**
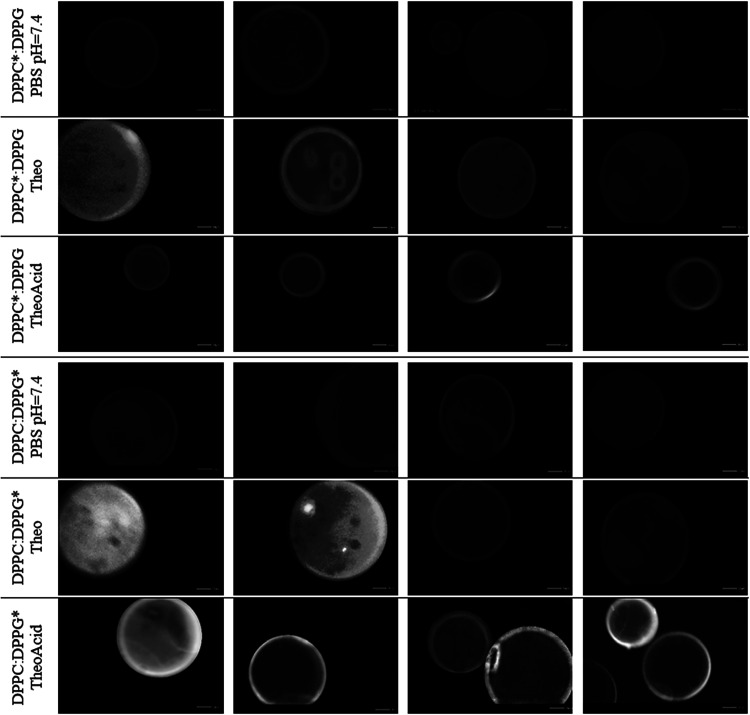
GUVs of the mixed DPPC:DPPG 8:2 model of pulmonary surfactant exposed to 10^–4^ mol/L theophylline and theophylline-7-acetic acid and visualized by fluorescence microscopy.

## Conclusions

In this work, we focus on providing a physicochemical explanation of the interfacial behavior of methylxanthines in contact with pulmonary surfactant models. The two representatives of methylxanthines, theophylline and theophylline-7-acetic acid were employed, while the developed models included DPPC, DPPG, and their 8:2 molar mixture to better reflect the actual lipidic composition of the pulmonary surfactant. By combining thermodynamic analysis with advanced meso- and nanoscale characterization at the air–water interface, we have demonstrated that these drugs, particularly the acidic derivative, are able to modulate the molecular organization and mechanical properties of the model phospholipid membranes at the air–water interface.

Langmuir isotherm analysis demonstrates that methylxanthines significantly reduce the surface stability of both single-component and mixed lipid monolayers, as evidenced by a marked decrease in the collapse pressure. The calculated compression modulus values further reveal that both theophylline and its acidic derivative fluidize the lipid matrix, with the latter inducing more substantial alterations to the film’s elastic properties. This is further evidenced by Brewster angle microscopy that methylxanthines induce significant fluidization of the DPPC matrix. Additionally, TheoAcid prevents the formation of regular crystalline domains, leading to a more disordered mesoscale structure. GIXD analysis also reveals a change in the 2D lattice of the DPPG monolayers. The presence of TheoAcid induces a shift from a rectangular to hexagonal organization, suggesting that the drug facilitates a more upright and compact arrangement of lipid molecules. PM-IRRAS data demonstrate that the interactions are primarily localized at the polar headgroup region involving carbonyl ester and especially phosphate groups and are of an electrostatic nature. More specifically, TheoAcid promotes the hydration of phosphates, which is most pronounced at lower surface pressures before the drug is partially expulsed at higher, physiologically relevant surface pressure, when the increased dehydration of phosphate groups by the drug takes place, which is also connected with the observed increased organization of the DPPG monolayer as shown by the GIXD results proving the change from rectangular to hexagonal arrangement. Methylxanthines also affect the dynamic reversibility of the layers, especially the mixed DPPC:DPPG models. In this case, the presence of methylxanthines leads to an increase in the free energy of hysteresis during cyclic compression–expansion, which suggests the formation of irreversible aggregates that may affect the ability of the surfactant to respread efficiently during the respiratory cycle. These results are also further confirmed by the observed bilayer reorganization using 3D models consisting of GUVs, where the drugs induce phase separation and morphological changes, including a reduction in the vesicle size under the influence of TheoAcid.

In summary, this multiscale investigation highlights the importance of electrostatic interactions and structural changes imposed by methylxanthines on the pulmonary interface. These results support the biophysical feasibility of inhalation-based delivery for methylxanthines, provided that the drug-induced alterations to surfactant integrity are carefully managed, e.g., by employing drug delivery systems suitable for inhalation administration.

## Supplementary Material



## Data Availability

Data are available in the data repository https://doi.org/10.58132/6QBXXA.

## Notes

Values of characteristic parameters for the isotherms and values of thermodynamic functions of hysteresis for DPPC, DPPG, and DPPC:DPPG 8:2 Langmuir monolayers formed on pure water subphase and water subphase containing methylxanthines; characteristic GIXD parameters of DPPC and DPPG Langmuir monolayers at 30 mN/m formed on pure water and water subphase containing theophylline-7-acetic acid; PM-IRRAS spectra of DPPG monolayers on pure water subphase and a water subphase containing methylxanthines in the CH spectral region; and PM-IRRAS band position for a polar headgroup region of DPPG monolayers formed on pure water subphase and subphase containing methylxanthines (PDF)
