# Supplementary material for: Interfacial Organization and Structural Changes in Model Lung Surfactants Induced by Methylxanthines
Source: Langmuir. 2026 Jun 4;42(23):17046–59. doi: 10.1021/acs.langmuir.6c01921 (PMC13309008; doi:10.1021/acs.langmuir.6c01921)
Supplement: Supplementary file 1 [file la6c01921_si_001.pdf]

SUPPORTING INFORMATION

Interfacial Organization and Structural Changes in Model Lung Surfactants Induced by Methylxanthines.

Wiktoria Kołomyjska[1], Michalina Zaborowska-Mazurkiewicz[2], Philippe Fontaine[3], Dorota Matyszewska[1*]

[1] *University of Warsaw, Faculty of Chemistry, Biological and Chemical Research Centre, Żwirki i Wigury 101, 02089 Warsaw, Poland*

[2] *University of Warsaw, Faculty of Chemistry, Pasteura 1, 02093 Warsaw, Poland*

[3] *Synchrotron Soleil, L'Orme des Merisiers, Départementale 128, Saint-Aubin 91190, France*

Corresponding author:
Email: dorota.matyszewska@chem.uw.edu.pl

Contents

Table S1 Values of characteristic parameters for the isotherms for DPPC, DPPG and DPPC:DPPG 8:2 Langmuir monolayers formed on pure water subphase and water subphase containing methylxanthines.

Table S2 Values of thermodynamic functions of hysteresis for DPPC, DPPG and DPPC:DPPG 8:2 Langmuir monolayers formed on pure water subphase and water subphase containing methylxanthines

Table S3 Characteristic GIXD parameters of DPPC and DPPG Langmuir monolayers at 30 mN/m formed on pure water and water subphase containing theophylline-7-acetic acid.

Fig. S1 PM-IRRAS spectra of DPPG monolayers on pure water subphase and a water subphase containing methylxanthines in the CH spectral region.

Table S4 PMIRRAS band position for a polar headgroup region of DPPG monolayers formed on pure water subphase and subphase containing methylxanthines.

Table S1 Characteristic parameters of DPPC, DPPG and DPPC:DPPG 8:2 Langmuir monolayers formed on pure water subphase and water subphase containing 10^{-4} mol/L concentrations of methylxanthines.

Lipid	$A_{\pi=10mN/m}$ / $Å^2$	$A_{\pi=30mN/m}$ / $Å^2$	A_{coll} / $Å^2$	π_{coll} / $mN\ m^{-1}$	Cs^{-1}_{max} / $mN\ m^{-1}$
			DPPC		
water	51.8± 0.4	41.9 ± 0.2	31.7 ± 0.3	72.0 ± 0.3	225 ± 11
10^{-4} mol/L Theo	47.3± 0.8	31.1 ± 0.5	19.2 ± 1.1	67.9 ± 1.6	205 ± 14
10^{-4} mol/L TheoAcid	55.6± 0.7	43.6 ± 0.7	37.2 ± 1.0	53.5 ± 2.4	195 ± 16
			DPPG		
water	45.2 ± 1.1	41.1 ± 0.3	33.3 ± 1.9	52.4 ± 1.5	485 ± 9
10^{-4} mol/L Theo	48.6 ± 0.6	43.1 ± 0.1	36.9 ± 0.1	49.0 ± 1.3	425 ± 9
10^{-4} mol/L TheoAcid	52.0 ± 1.4	36.6 ± 0.1	35.1 ± 2.5	46.2 ± 1.5	310 ± 8
			DPPC:DPPG 8:2		
water	41.1 ± 0.8	38.1 ± 0.8	29.9 ± 0.7	61.2 ± 6.1	236 ± 10
10^{-4} mol/L Theo	43.4 ± 0.5	34.7 ± 0.4	29.3 ± 0.4	60.2 ± 0.1	201 ± 1
10^{-4} mol/L TheoAcid	50.4 ± 0.8	36.4 ± 0.4	29.9 ± 0.1	61.6 ± 1.5	224 ± 3

Table S2 Thermodynamic functions of hysteresis: the free energy of compression (ΔG_{comp}), expansion (ΔG_{exp}) and hysteresis (ΔG^{hys}), the configurational entropy of hysteresis ($T\Delta S^{hys}$), and the enthalpy of hysteresis (ΔH^{hys}) calculated between π=1 mN/m and π=30 mN/m for DPPC, DPPG and DPPC:DPPG 8:2 Langmuir monolayers formed on pure water subphase and water subphase containing 10^{-4} mol/L concentrations of methylxanthines.

Subphase	ΔG_{comp} / kcal mol^{-1}	ΔG_{exp} / kcal mol^{-1}	ΔG^{hys} / kcal mol^{-1}	$T\Delta S^{hys}$ / kcal mol^{-1}	ΔH^{hys} / kcal mol^{-1}
			DPPC		
water	0.48 ± 0.01	0.44 ± 0.01	-0.04 ± 0.01	-0.17 ± 0.01	-0.21 ± 0.01
10^{-4} mol/L Theo	0.56 ± 0.02	0.51 ± 0.02	-0.05 ± 0.01	-0.24 ± 0.01	-0.28 ± 0.01
10^{-4} mol/L TheoAcid	0.59 ± 0.02	0.49 ± 0.02	-0.11 ± 0.01	-0.53 ± 0.02	-0.64 ± 0.03
			DPPG		
water	0.18 ± 0.02	0.17 ± 0.02	-0.01 ± 0.01	-0.04 ± 0.02	-0.05 ± 0.02
10^{-4} mol/L Theo	0.17 ± 0.01	0.16 ± 0.01	-0.01 ± 0.01	-0.02 ± 0.01	-0.03 ± 0.01
10^{-4} mol/L TheoAcid	0.25 ± 0.07	0.19 ± 0.01	-0.06 ± 0.06	-0.36 ± 0.04	-0.41 ± 0.09
			DPPC:DPPG 8:2		

water	0.25 ± 0.01	0.22 ± 0.01	-0.03 ± 0.01	-0.24 ± 0.01	-0.26 ± 0.03
10^{-4} mol/L Theo	0.31 ± 0.03	0.26 ± 0.03	-0.05 ± 0.01	-0.32 ± 0.02	-0.37 ± 0.02
10^{-4} mol/L TheoAcid	0.40 ± 0.03	0.28 ± 0.02	-0.12 ± 0.01	-0.89 ± 0.04	-1.00 ± 0.05

Table S3 Characteristic GIXD parameters of DPPC and DPPG Langmuir monolayers at 30 mN/m formed on pure water subphase and water subphase containing 10^{-4} mol/L of theophylline-7-acetic acid.

Subphase	Q_{xy} / Å^{-1}	Q_z / Å^{-1}	L_{xy} / Å	*d* / Å	*a,b* (Å)	γ (°)	τ (°)	A_{uc} / Å^2
				DPPC				
water	<0,2> 1.464	0	533	4.29	5.331;	90	26.78	45.76
	<-1,1> 1.387	0.59	92	4.53	8.584			
10^{-4} mol/L TheoAcid	<0,2> 1.464	0	371	4.29	5.387;	90	28.69	46.23
	<-1,1> 1.377	0.64	72	4.56	8.581			
				DPPG				
water	<0,2> 1.495	0	592	4.20	4.919;	90	9.4	41.34
	<-1,1> 1.480	0.21	184	4.25	8.403			
10^{-4} mol/L TheoAcid	1.501	0	384	4.18	4.830	120	-	20.20

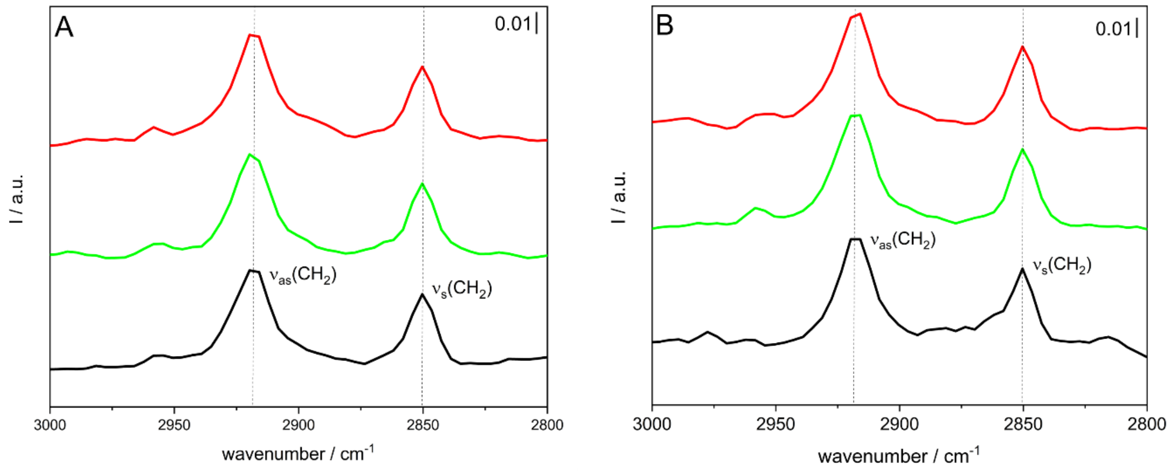

Fig. S1 PM-IRRAS spectra of DPPG monolayers compressed to A) 10 mN/m; B) 30 mN/m on pure water subphase (black) and a water subphase containing 10^{-4} mol/L solution of theophylline (green) and theophylline-7-acetic acid (red) in the ~3000 cm^{-1} to ~2800 cm^{-1} spectral region (acyl chain).

Table S4 PMIRRAS band position (in cm^{-1}) for a polar headgroup region of DPPG monolayers formed on pure water subphase and subphase containing 10^{-4} mol/L theophylline and theophylline-7-acetic.

Subphase	H_2O	10^{-4} *mol/L Theo*	10^{-4} *mol/L TheoAcid*
		10 mN/m	
$\nu(C{=}O)$	1739<br>1724	1739<br>1728	1739<br>1728
$\nu_{as}(PO_2^-)$	1218	-	1222<br>1207
$\nu_s(PO_2^-)$	1107<br>1095<br>1052	1107<br>1083<br>1045	1103<br>1087-1079<br>1041
		30 mN/m	
$\nu(C{=}O)$	1739<br>1728	1743<br>1728	1743<br>1728
$\nu_{as}(PO_2^-)$	1238	1238	1238
$\nu_s(PO_2^-)$	1111<br>1068<br>1049	1107<br>1087-1080<br>1045	1107<br>1087<br>1064<br>1038
